# Clinical, Laboratory and Anatomopathological Findings of an Outbreak of Conidiobolomycosis in Sheep in the State of Rio Grande do Norte, Brazil

**DOI:** 10.3390/ani16081231

**Published:** 2026-04-17

**Authors:** Carlos Alberto Queiroz de Aquino, Geovana Kelly dos Santos Ribeiro, Ruan da Cruz Paulino, Laynaslan Abreu Soares, Yanca Góes dos Santos Soares, Jael Soares Batista, Francisco Marlon Carneiro Feijó, Jefferson Filgueira Alcindo

**Affiliations:** 1 Department of Animal Science, Universidade Federal Rural do Semi-Árido, Mossoró 59625-900, RN, Brazil; carlos.aqno16@gmail.com (C.A.Q.d.A.); geovana.ribeiro@alunos.ufersa.edu.br (G.K.d.S.R.); jael.batista@ufersa.edu.br (J.S.B.); marlon@ufersa.edu.br (F.M.C.F.); jefferson.alcindo@yahoo.com.br (J.F.A.); 2 Rural Health and Technology Center, Universidade Federal de Campina Grande, Patos 58708-110, PB, Brazil; laynaslanabreu@gmail.com (L.A.S.); yancagoes@hotmail.com (Y.G.d.S.S.)

**Keywords:** Brazil, *Conidiobolus*, nasal cavity, fungus, granuloma, sheep farming

## Abstract

Diseases of the nasal cavity have a diverse etiology and cause severe disorders in animals. Conidiobolomycosis is a type of zygomycosis caused by the fungus *Conidiobolus* spp., occurring more frequently in sheep. The objective of this study is to describe the clinical and epidemiological characteristics, as well as microbiological and histopathological findings, of an outbreak of conidiobolomycosis in sheep. A total of 12 animals out of a herd of 70 were affected, representing a morbidity of 17.1%; mortality and lethality were 11.4% (8/70) and 66.6% (8/12), respectively, of which 4 sheep died spontaneously and 4 were euthanized for diagnostic purposes due to the severity of the clinical condition. Necropsy was performed on 4 (33.3%) of the 12 affected animals, and after opening the skull in the sagittal section, a friable mass with coloration ranging from whitish-yellow to gray-green, as well as areas of necrosis, were evident in regions such as the nasal meatus, conchae, nasopharynx, hard palate, cribriform plate, meninges, and frontal lobe of the brain. *Conidiobolus lamprauges* was isolated from samples collected from 6 animals from nasal discharge, fungal granuloma, and intranasal swab. Reproductive structures consistent as kind of zygospores with *C. lamprauges* and *C. incongruus* were also identified. Regarding the clinical form, animals 1, 2, 3, and 4 presented manifestations of the nasopharyngeal form, with the first three progressing to the rhinocerebral form; in animal 5, the clinical form found was rhinofacial. It is concluded that clinical signs may vary with the presentation of the disease as well as the involved agent. Early diagnostic alternatives such as fungal isolation from material collected from intranasal swabs can be useful and employed in affected herds, enabling earlier intervention.

## Simple Summary

Conidiobolomycosis is a fungal disease affecting sheep and is associated with a high mortality rate. This report describes the epidemiological, clinical, laboratory, and pathological findings of conidiobolomycosis in an outbreak diagnosed in northeastern Brazil. We emphasize the importance of early diagnostic techniques and provide a detailed description of pathological lesions, which is crucial in cases like this.

## 1. Introduction

Conidiobolomycosis is a fungal disease caused by fungi of the genus *Conidiobolus* (Entomophthorales, Ancylistaceae) [1]. The main species reported to cause disorders in animals are *C. lamprauges*, *C. coronatus*, and *C. incongruus*, primarily affecting sheep, horses, dogs, goats, and immunosuppressed humans [2]. The disease has already been reported in some Brazilian states caused by *C. coronatus* and *C. lamprauges* [2,3,4,5,6,7,8,9,10,11]. There are no records of conidiobolomycosis caused by *C. incongruus* in Brazil. Two clinical forms are reported for the disease: rhinopharyngeal, the most common affecting mainly the ethmoidal region, and rhinofacial, less common affecting the nasal vestibule and the skin of the nostril [10]. Clinical signs of both forms include nasal discharge, fever, lethargy, anorexia, weight loss, and dyspnea [5,7,10].

These fungi are commonly found in tropical and subtropical regions where they find ideal conditions of humidity and temperature for development. However, they can also be found in semi-arid regions, especially in places where microclimates conducive to their development are formed, such as dam margins, streams, and brooks, especially those with aquatic plants, as well as areas with high amounts of decomposing organic matter [3]. Sheep appear to be the most susceptible species to the fungus. This species is primarily affected due to its grazing habit close to the ground, increasing the possibility of inhaling conidia present in vegetation and decomposing organic matter [12].

The diagnosis of the disease is made through clinical and histopathological findings, associated with the culture and isolation of the etiological agent, and may also involve the identification of fungal genetic material or antigenic studies, using the Polymerase Chain Reaction (PCR) and Immunohistochemistry (IH) techniques [9]. Late diagnosis and rapid disease progression contribute to this lethality, as animals with advanced lesions may not respond to treatment [10]. Early diagnostic methods could be useful for identifying carrier animals or lesions in the initial phase; however, research for this purpose has not been conducted.

More studies are needed to refine techniques and fill gaps to better elucidate the pathogenesis and spread of the disease, and this work could serve as the basis for that. The present study aims to describe the epidemiological, clinical, and diagnostic findings of an outbreak of conidiobolomycosis in sheep in the state of Rio Grande do Norte, contributing new data regarding the disease and the possibility of in vivo diagnosis.

## 2. Materials and Methods

The outbreak occurred in the municipality of José da Penha (Sitio Catolezinho, 680, 59987-000. Latitude: between 06°16′57.44″ S and 06°17′30.05″ S; longitude: between 38°16′09.78″ W and 38°16′27.62″ W), RN, on a farm with 70 animals, crossbreeds of Santa Inês and Cariri, between the months of January and May 2023. Information about the animals, disease progression, and epidemiological aspects were collected from the owner by completing individual animal records and assessing herd characteristics. Out of the total number of animals, 12 presented the disease, and 10 underwent physical evaluation. Blood samples (10 mL/animal) were collected from six animals by venipuncture (Animals 01, 02, 03, 04, 05, and 06) for hematological analysis.

Euthanized sheep were necropsied, and tissue samples from the ethmoidal and nasopharyngeal regions, lungs, and brain were collected. The samples were fixed in 10% formalin for subsequent histological study, using hematoxylin-eosin (HE) staining, Grocott’s methenamine silver (GMS) stain, and Periodic Acid-Schiff (PAS) stain [12]. Nasal secretion samples (Animals 1 and 2) and masses found after skull opening (Animals 1, 2, 3, and 4) were sent for microbiological culture. In addition, a sterile long-shaft swab was inserted into the nostril corresponding to the macroscopically observed lesion in six animals (animals 3, 4, 5, 6, 7, and 8), and the collected samples were subsequently inoculated onto culture media. Each sample was individually inoculated on blood agar, MacConkey agar, and Sabouraud dextrose agar supplemented chloramphenicol [13].

Unfortunately, given the circumstances of access, availability of equipment and distance from university property, other diagnostic strategies, especially imaging tests, could not be used.

## 3. Results

### 3.1. Epidemiological Data

The morbidity, mortality, and case fatality rates were 17.1% (12/70), 11.4% (8/70), and 66.6% (8/12), respectively. Among the deceased, four sheep died spontaneously and four were euthanized for diagnostic purposes due to the severity of their clinical condition (Table 1). The remaining affected animals, which neither died spontaneously nor were euthanized, were isolated on the property and monitored by the owner over the course of a year up to the time of this report. The affected sheep ranged in age from approximately eight months to four years and were raised in a floodplain area with native vegetation, predominantly grasses. The owner reported that part of the area was submerged or at least partially flooded during certain times of the year due to water reservoir overflow. Additionally, the cases occurred between January and May, months with the highest rainfall in the region, averaging approximately 700 mm of precipitation during this period. During a visit to the property, it was observed that the sheep preferred drinking water from a stream area covered with aquatic plants. Another important aspect identified was the accumulation of mechanically removed aquatic plants near the handling pen, where the sheep were kept overnight, obstructing the route between the grazing area and the pen.

### 3.2. Clinical Signs and Hematological Findings

The clinical signs observed varied according to the presentation of the disease and are shown in Table 2. Sheep 1, 2, 3, and 4 exhibited the nasopharyngeal form, with the first three progressing to the rhinocerebral form. Sheep 5 showed the rhino-facial form, and sheep 6, 7, and 8 were also diagnosed with the nasopharyngeal form.

In the hemograms of six affected sheep, normocytic normochromic non-regenerative anemia, leukocytosis with neutrophilia and a left shift, and inversion of the lymphocyte/neutrophil ratio were observed.

### 3.3. Pathological Findings

**Figure 1:**
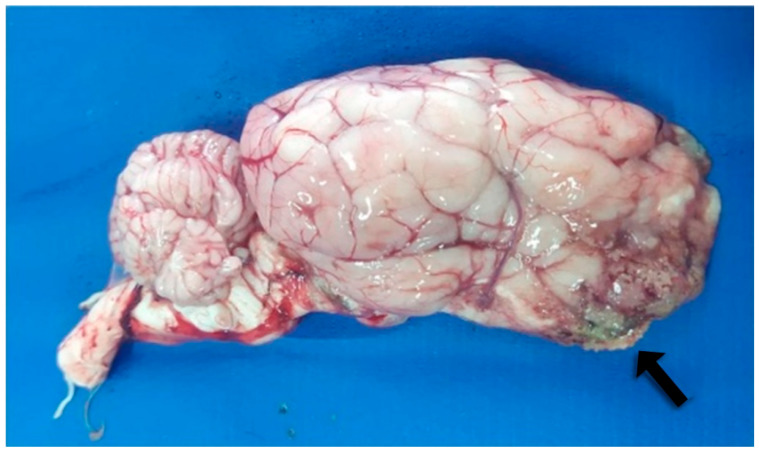
Frontal cortex of Sheep 1 showing an extensive focal area of malacia (black arrow).

In four necropsied sheep, an enlargement of the nasal region was observed. On longitudinal sectioning of the head, friable, necrotic, yellowish-gray masses were identified, extending through the nasal conchae and meatuses, particularly involving the ethmoidal region and turbinate bones, nasopharynx, hard and soft palate, cribriform plate, meninges, and frontal lobe of the brain (Figure 1). Serial transverse sections of the brain revealed a focal, granular, darkened area in the frontal cortex. Additionally, the regional lymph nodes were enlarged and exhibited an irregular cut surface. In one sheep, multiple small nodules were also present in the pulmonary parenchyma.

**Figure 2:**
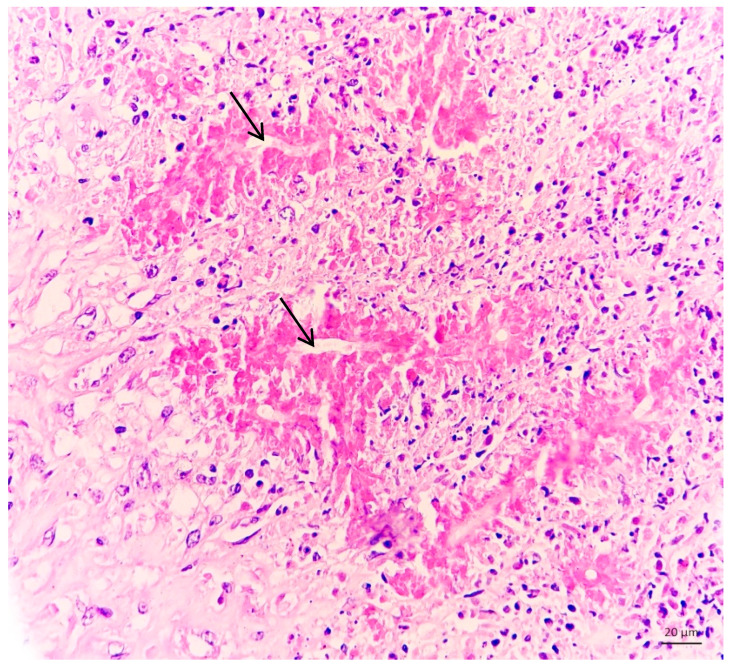
Histological appearance of conidiobolomycosis in the nasal cavity of a sheep. Note the inflammatory infiltrate associated with negative images of hyphae (black arrows) surrounded by radiated eosinophilic material (Splendore-Hoeppli reaction) HE. 20×.

Histopathological examination revealed pyogranulomatous rhinitis associated with negatively stained hyphae, characterized by multifocal to coalescing areas of necrosis permeated by a dense inflammatory infiltrate composed of macrophages, lymphocytes, plasma cells, neutrophils, and multinucleated giant cells, along with proliferation of fibrous connective tissue. Within the necrotic areas and inflammatory infiltrates, tubular, negatively stained hyphal profiles, in both longitudinal and transverse sections, were observed. These hyphae were surrounded by lightly radiated, intensely eosinophilic material, consistent with a Splendore-Hoeppli reaction (Figure 2).

Some hyphae contained amorphous, lightly eosinophilic material within their cytoplasm and were also detected in the cytoplasm of multinucleated giant cells, occasionally exhibiting bulbous dilations filled with finely granular basophilic material. PAS staining highlighted hyphae with lightly pink-stained walls and bulbous terminal expansions. GMS staining revealed hyphae with strongly black-impregnated, irregular walls, rarely septate and branched, with multiple dilations. The hyphae measured 8–22 µm in diameter, consistent with fungi of the genus *Conidiobolus* spp.

**Figure 3:**
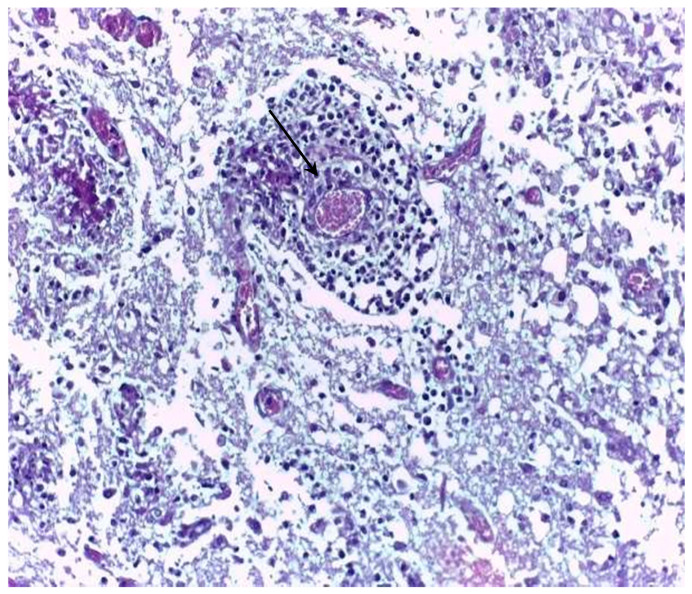
Histological appearance of conidiobolomycosis in a sheep. Note vasculitis (black arrow). HE. 40×.

In the frontal cortex of the brain, marked thickening of the leptomeninges due to a pronounced pyogranulomatous inflammatory infiltrate was observed. Hyphae were seen within the cytoplasm of giant cells and amidst the Splendore-Hoeppli reaction in the leptomeninges. In the adjacent neuropil, a focal extensive area of malacia with gitter cells and moderate inflammatory infiltrate composed of lymphocytes, plasma cells, macrophages, and rare neutrophils, along with vasculitis, evidenced by the presence of mononuclear inflammatory cells within the vessel walls (Figure 3).

**Figure 4:**
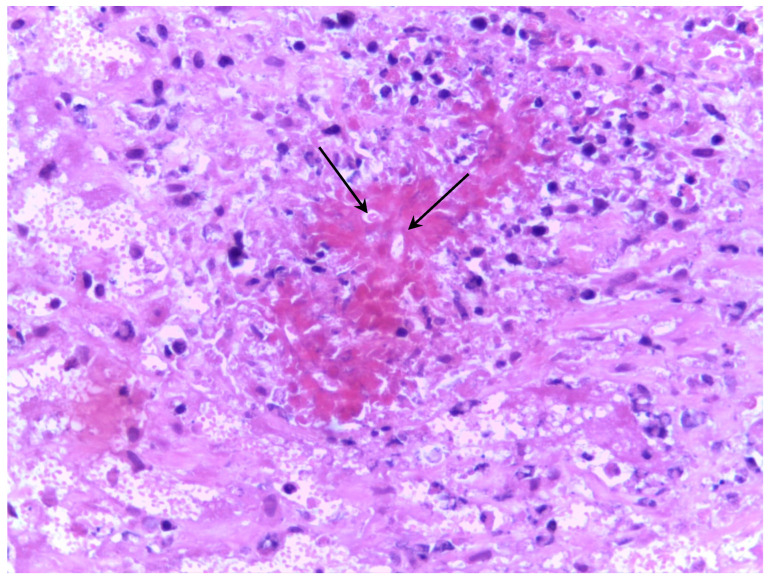
Histological appearance of conidiobolomycosis in a sheep. Note multifocal areas of eosinophilic material (Splendore-Hoeppli reaction) with negative images of hyphae surrounded by inflammatory cell infiltrates in lung tissue indicated by the arrows. HE. 40×.

In the lung tissue, connective tissue encapsulating multifocal areas of eosinophilic material (Splendore-Hoeppli reaction) with negative images of hyphae surrounded by inflammatory cell infiltrates predominantly composed of macrophages, lymphocytes, plasma cells, and fewer neutrophils was observed, as detailed in Figure 4.

### 3.4. Microbiological Findings

Fungal culture samples were collected from eight sheep. The collection methods varied, including material from the fungal mass after skull opening, external nasal discharge, and/or long swab intranasal samples (Table 3).

**Figure 5:**
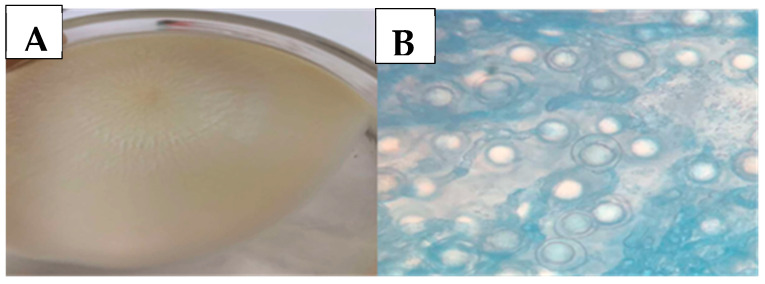
Microbiological culture of samples from the cavities of sheep with conidiobolomycosis. (**A**) Sparse, smooth, and white colony after three days. (**B**) Zygospores with a single large homogeneous internal globule—100× magnification.

Fungal growth was observed in samples from six sheep. Macroscopically, the colonies showed powdery structures with short aerial mycelia, radiating from the center. At 100× magnification, reproductive fungal structures characteristic of *Conidiobolus lamprauges* were observed, showing mature zygospores with a single large homogeneous globule measuring 12 to 18 µm (Figure 5) [14].

## 4. Discussion

The mortality and morbidity rates observed are consistent with those reported in other studies, including those conducted in the semi-arid region of Brazil [3,15,16]. The clinical and epidemiological findings, along with histopathological changes and pathogen isolation, were critical for diagnosing this outbreak. The presence of aquatic plants and abundant organic matter in the grazing areas seems to have also contributed to the development of the disease [3]. The presence of floodplain areas on the property, with humidity and temperature favorable for fungal growth, combined with the grazing patterns of the sheep, likely influenced the relatively high morbidity observed in the flock.

Another factor that appears to contribute to the increase in cases is the mechanical removal of aquatic plants, as this action may have facilitated access to spores for the animals, either through greater dispersion by mechanical action along the animals’ path or by bringing decomposing material into areas where the sheep congregate. The occurrence of conidiobolomycosis in sheep raised under similar environmental conditions, with grazing areas bordered by a river and partially flooded, was reported in a similar study [17].

Regarding other epidemiological factors, there was no interaction with the sex, age, or physiological status of the animals; however, as in most cases reported in the country, the affected animals were of the Santa Inês breed. It cannot be affirmed that there is a racial predisposition, as the higher incidence in this breed may be associated with the fact that the Santa Inês breed is one of the most commonly used in farming, both for purebred maintenance and for crossbreeding [15].

Fungal isolation was successfully achieved from nasal discharge, fungal granuloma, and intranasal swab. The latter has not been previously reported in the literature. The protocol used for sampling and isolating the etiological agent proved effective; however, some caveats should be noted. Firstly, the nasal cavity is naturally colonized by various microorganisms, so sample culture should always be performed on media supplemented with antimicrobial agents to inhibit bacterial growth [10]. Additionally, in some cases, more than one fungal colony may grow, necessitating the isolation of all colonies with distinct macroscopic characteristics. Microscopically, fungi can be differentiated by the type of zygospores. *C. lampragues* can be identified by having only a single globule inside the structure [14].

The use of a long-handled intranasal swab for microorganism isolation proved efficient, as in all cases where isolation was achieved using other methods and this method was employed, the etiological agent was also isolated. Furthermore, this method allows for in vivo diagnosis, as current literature only records post-mortem isolation. The disadvantage of this technique is potential area contamination; however, it can be an extremely useful screening and isolation method, requiring low-cost materials and being easy to perform.

Macroscopic and histopathological evaluation of lesions, coupled with clinical signs, allowed for the diagnosis of both clinical forms of the disease. Other reports have also revealed the occurrence of both presentations [15,16], although the presence of only one form is more common. Coincidentally, one of these outbreaks occurred in the same state where this case was diagnosed. As observed in this outbreak, the nasopharyngeal form predominated in cases where *C. lamprauges* was isolated [3,16,17]. There may be a correlation between the clinical form and the *Conidiobolus* species, but further studies are needed in this regard.

It is important to perform a differential diagnosis of conidiobolomycosis in relation to other fungal infections, which trigger eosinophilic granulomas, with the Splendore-Hoeppli phenomenon around hyphae, such as entomophthoromycosis, caused by pathogenic species of the order Entomophthorales. Although the Hoeppli phenomenon is a histopathological finding shared by infections caused by these groups of pathogens, the pathogenic species of the order Entomophthorales cause infections restricted to subcutaneous tissues but can be involved in intestinal and disseminated infections [18], whereas conidiobolomycosis caused by *Pythium insidiosum* manifests as an infection that originates in the paranasal sinuses, resulting in chronic granulomatous rhinitis that frequently spreads to other regions, causing nasopharyngeal and rhinocerebral lesions [3].

In three out of four necropsied sheep, there was nervous system involvement due to fungal invasion through the cribriform plate, characterizing the rhinocerebral form [14,15]. These cases primarily present with the nasopharyngeal form, which, due to fungal dispersion and penetration, evolves into the rhinocerebral form [10]. However, this study observed that two animals presented with the rhinocerebral form of the disease even without a fungal granuloma in the nasopharynx. Thus, it may be worth considering a third classification for the clinical forms of the disease, recognizing the rhinocerebral form as a standalone possibility [17], rather than merely an evolution of the nasopharyngeal form.

The hematological alterations found are consistent with those reported in other outbreaks of the disease [16,19]. Anemia may have occurred due to nutritional deficiency and/or nasal hemorrhage [19]. Additionally, leukocytosis with a left shift has been observed in inflammatory and infectious processes of fungal origin [19]. More repetitions and refinement of the techniques and procedures described in this report will be necessary. However, the demonstrated efficacy of the adopted protocols presents an interesting possibility for early diagnosis and intervention in sheep with conidiobolomycosis.

## 5. Conclusions

It is concluded that clinical signs may vary with the presentation of the disease as well as the involved agent. Early diagnostic alternatives such as fungal isolation from material collected from intranasal swabs can be useful and employed in affected herds, enabling earlier intervention. Further studies are still needed to refine the techniques and fill gaps in order to better elucidate the pathogenesis and dissemination of the disease, and this work could be one of the initial steps towards that.

## Figures and Tables

**Table 1:** Clinical presentation, treatment and outcome of affected sheep.

Animal	Sex	Age	Outcome	Treatment *	Presentation
1	Female	11 months	Euthanized	No	Nasopharyngeal
2	Male	13 months	Euthanized	No	Nasopharyngeal
3	Female	26 months	Euthanized	No	Nasopharyngeal
4	Female	18 months	Euthanized	Yes	Nasopharyngeal
5	Female	32 months	Recovery	Yes	Rhino-facial
6	Male	8 months	Slaughter	No	Nasopharyngeal
7	Male	14 months	Slaughter	No	Nasopharyngeal
8	Male	13 months	Slaughter	No	Nasopharyngeal
9	Male	28 months	Spontaneous died	No	Rhino-facial
10	Female	21 months	Spontaneous died	No	Nasopharyngeal
11	Female	43 months	Spontaneous died	No	Rhino-facial
12	Male	15 months	Spontaneous died	No	Rhino-facial

* Potassium iodide, 1 g/day, intravenously, once daily, for 15 days.

**Table 2:** Clinical signs presented in sheep diagnosed with conidiobolomycosis.

Animal	Clinical Sign						
	Changes in Lymph Nodes	Exophthalmos	Dyspnea	Apathy	Blindness	Nasal Discharge	Increased Volume in the Nasal Region
1	P	P	P	P	P	P	A
2	P	P	P	P	P	P	A
3	P	P	P	P	P	P	A
4	P	A	P	P	A	P	A
5	P	A	P	P	A	P	P
6	P	A	A	A	A	A	A
7	P	A	A	A	A	A	A
8	P	A	A	A	A	A	A
9	P	A	P	P	A	P	P
10	P	P	P	P	P	P	A
11	P	A	P	P	A	P	P
12	P	A	P	P	A	P	P

P: present; A: absent.

**Table 3:** Fungal growth according to the sample collection method from eight sheep with conidiobolomycosis.

Animal	Collection Method		
	Intranasal	Fungal Mass	Secretion
1	NE	P	A
2	NE	P	A
3	P	P	NE
4	P	P	NE
5	P	NE	P
6	A	NE	NE
7	P	NE	NE
8	A	NE	NE

P: present; A: absent.; NE: not evaluated.

## Data Availability

The original contributions presented in this study are included in the article/Supplementary Material. Further inquiries can be directed to the corresponding author.

## Supplementary Materials

The following supporting information can be downloaded at: https://www.mdpi.com/article/10.3390/ani16081231/s1.
